# Aromatic secondary metabolite production from glycerol was enhanced by amino acid addition in *Pichia pastoris*

**DOI:** 10.1007/s00253-023-12798-5

**Published:** 2023-09-27

**Authors:** Ryota Kumokita, Takanobu Yoshida, Tomokazu Shirai, Akihiko Kondo, Tomohisa Hasunuma

**Affiliations:** 1 Graduate School of Science, Technology and Innovation, Kobe University, 1-1 Rokkodai, Nada, Kobe, 657-8501 Japan; 2 RIKEN Center for Sustainable Resource Science, 1-7-22 Suehiro, Tsurumi, Yokohama, 230-0045 Japan; 3 Engineering Biology Research Center, Kobe University, 1-1 Rokkodai, Nada, Kobe, 657-8501 Japan

**Keywords:** Aromatic secondary metabolites, *Pichia pastoris*, Glucose, Glycerol, Amino acid, Metabolomics

## Abstract

**Abstract:**

Aromatic secondary metabolites are widely used in various industries, including the nutraceutical, dietary supplement, and pharmaceutical industries. Their production currently relies on plant extraction. Microbe-based processes have recently attracted attention as sustainable alternatives to plant-based processes. We previously showed that the yeast *Pichia pastoris* (*Komagataella phaffii*) is an optimal host for producing aromatic secondary metabolites. Additionally, titers of resveratrol, an aromatic secondary metabolite, increased by 156 % when glycerol was used as a carbon source instead of glucose. However, the mechanisms by which glycerol resulted in higher production has remained unclear. In this study, we aimed to elucidate how *P. pastoris* produces higher levels of aromatic secondary metabolites from glycerol than from glucose. Titers of *p*-coumarate, naringenin, and resveratrol increased by 103 %, 118 %, and 157 %, respectively, in natural complex media containing glycerol compared with that in media containing glucose. However, the titers decreased in minimal synthetic medium without amino acids, indicating that *P. pastoris* cells used the amino acids only when glycerol was the carbon source. Fermentation with the addition of single amino acids showed that resveratrol titers from glycerol varied depending on the amino acid supplemented. In particular, addition of aspartate or tryptophan into the medium improved resveratrol titers by 146 % and 156 %, respectively. These results suggest that *P. pastoris* could produce high levels of aromatic secondary metabolites from glycerol with enhanced utilization of specific amino acids. This study provides a basis for achieving high-level production of aromatic secondary metabolites by *P. pastoris*.

**Key points:**

**•** *P. pastoris can produce high levels of aromatic metabolites from glycerol*

**•** *P. pastoris cells use amino acids only when glycerol is the carbon source*

**•** *Aromatic metabolite titers from glycerol increase with amino acids utilization*

**Supplementary Information:**

The online version contains supplementary material available at 10.1007/s00253-023-12798-5.

## Introduction

Aromatic secondary metabolites have a wide range of applications, from nutraceuticals and dietary supplements to pharmaceuticals, and represent a multi-billion-dollar global market (Kallscheuer et al. 2019; Cao et al. 2020; Gu et al. 2020; Xu et al. 2020; Braga and Faria 2022). Given their complex structures, these compounds are difficult to synthesize from petrochemicals, and their production currently relies on extraction from plants (Facchini et al. 2012; Pyne et al. 2019). However, extraction from plants cannot meet the large demands for these compounds because of complex purification procedures, low yields, and long plant growth times (Suástegui and Shao 2016; Xu et al. 2020). As alternatives to plant extraction, microbe-based processes have attracted considerable attention for producing aromatic secondary metabolites by heterologous reconstitution of plant genes into microorganisms and for building a more sustainable society (Cravens et al. 2019; Yuan and Alper 2019; Gong et al. 2022; Joshi and Mishra 2022; Jang et al. 2023).

To date, various microorganisms, such as *Escherichia coli* (Stahlhut et al. 2015), *Corynebacterium glutamicum* (Kogure and Inui 2018), *Saccharomyces cerevisiae* (Liu et al. 2019; Chrzanowski 2020), *Yarrowia lipolytica* (Gu et al. 2020; Larroude et al. 2021), and *Pichia pastoris* (*Komagataella phaffii*) (Kumokita et al. 2022), have been used as microbial hosts for producing aromatic secondary metabolites. Among these microorganisms, eukaryotic yeasts, including *S. cerevisiae*, *Y. lipolytica*, and *P. pastoris*, have proven to be attractive hosts because of their low susceptibility to phage contamination, robust cell growth, and high tolerance to high concentrations of metabolites (Krivoruchko and Nielsen 2015; Gu et al. 2020; Patra et al. 2021). In particular, *P. pastoris*, also known as the “biotech yeast”, has recently attracted considerable attention as a next-generation cell factory for synthetic biology, with the goal of generating valuable microbe-based products through metabolic engineering (Bernauer et al. 2021; Carneiro et al. 2022; Shrivastava et al. 2023).

However, the culture conditions, including carbon sources and medium compositions, suitable for the production of aromatic secondary metabolites by *P. pastoris* have not been investigated. In a previous study, we constructed a chassis strain optimized for tyrosine production in *P. pastoris* and achieved de novo microbe-based production of tyrosine-derived aromatic secondary metabolites, including norcoclaurine, reticuline, naringenin, and resveratrol, by *P. pastoris* (Kumokita et al. 2022). In addition, we found that resveratrol titers increased by 156 % after 96 h of fermentation with glycerol instead of glucose (Kumokita et al. 2022). However, the mechanisms by which glycerol, as a carbon source, improved the resveratrol titers in *P. pastoris* remained unclear.

Thus, in this study, we aimed to investigate how *P. pastoris* produces higher levels of aromatic secondary metabolites from glycerol than it does from glucose. The titers of several aromatic secondary metabolites, including *p*-coumarate, naringenin, and resveratrol, were measured in *P. pastoris* grown with different carbon sources (glucose or glycerol). The production of aromatic secondary metabolites in the presence or absence of amino acids as carbon sources was also studied. A comprehensive time-course analysis of the intracellular and extracellular metabolites during fermentation was performed during cultivation with glucose or glycerol. Based on the metabolomics results, we performed fermentation with addition of single amino acids and discussed the mechanism through which *P. pastoris* produces high levels of aromatic secondary metabolites from glycerol. The results of this study may serve as a basis for further research on the production of aromatic secondary metabolites by *P. pastoris*.

## Material and methods

### Plasmids, strains, and culture media

All plasmids and yeast strains used in this study are listed in Table 1. Plasmids were constructed following previously described methods (Kumokita et al. 2022).

**Table 1:** Yeast strains and plasmids

Strains	Description	Source
CBS7435	Wild-type (NRRL Y-11430 or ATCC 76273)	ATCC
CBS7435 *Δdnl4 Δhis4* (Control)	CBS7435 / *Δdnl4 Δhis4* :: *ADE1*	(Ito et al. 2018)
TAL	CBS7435 *Δdnl4 Δhis4* / pPGP-TAL	This study
T-ARO47m	TAL /pPGPH-ARO4^K229L^-ARO7^G141S^ [G418^r^, Hyg^r^]	This study
T4V-ARO47m	CBS7435 *Δdnl4 Δhis4* / pPGP-TAL-4CL-VST, pPGPH-ARO4^K229L^-ARO7^G141S^ [G418^r^, Hyg^r^]	(Kumokita et al. 2022)
T4CC-ARO47m	CBS7435 *Δdnl4 Δhis4* / pPGP-TAL-4CL, pPGPZ-CHS-CHI, pPGPH-ARO4^K229L^-ARO7^G141S^ [G418^r^, Zeo^r^, Hyg^r^]	(Kumokita et al. 2022)
Plasmids		
pPGP-TAL	G418^r^, *P*_*gap*_-*HaTAL*-*T*_*aox1*_	(Kumokita et al. 2022)
pPGPH-ARO4^K229L^-ARO7^G141S^	Hyg^r^, *P*_*gap*_-*ARO4*^*K229L*^ -*T*_*aox1*_, *P*_*gap*_-*ARO7*^*G141S*^-*T*_*aox1*_	(Kumokita et al. 2022)
pPGP-TAL-4CL-VST	G418^r^, *P*_*gap*_-*HaTAL*-*T*_*aox1*_, *P*_*gap*_-*At4CL*-*T*_*aox1*_, *P*_*gap*_-*VvVST*-*T*_*aox1*_,	(Kumokita et al. 2022)
pPGP-TAL-4CL	G418^r^, *P*_*gap*_-*HaTAL*-*T*_*aox1*_, *P*_*gap*_-*At4CL*-*T*_*aox1*_,	(Kumokita et al. 2022)
pPGPZ-CHS-CHI	Zeo^r^, *P*_*gap*_-*HaCHS*-*T*_*aox1*_, *P*_*gap*_-*MsCHI*-*T*_*aox1*_,	(Kumokita et al. 2022)

The parental *P. pastoris* strain used in this work was CBS7435 *Δdnl4 Δhis4*, derived from the CBS7435 strain (NRRL Y-11430 or ATCC 76273). *P. pastoris* was transformed using a lithium acetate-based method as previously reported (Ito et al. 2018). The *p*-coumarate-producing strain TAL was constructed as follows: the pPGP-TAL plasmid was linearized with *Aat*II, transfected into the CBS7435 *Δdnl4 Δhis4* strain, and then integrated into the *CCA38743* locus of its genomic DNA by single-crossover recombination. The pPGPH-ARO4^K229L^-ARO7^G141S^ plasmid was linearized with *Bsr*GI, transfected into the TAL strain, and then integrated into the *Arg4* locus of its genomic DNA by single-crossover recombination to generate the T-ARO47m strain. Details about the construction of the T4V-ARO47m and T4CC-ARO47m strains can be found in our previous work (Kumokita et al. 2022).

*P. pastoris* cells were grown in SD medium [6.7 g/L yeast nitrogen base without amino acids (YNB), 80 mg/L histidine, and 20 g/L glucose], SG medium (6.7 g/L YNB, 80 mg/L histidine, and 20 g/L glycerol), SCD medium [6.7 g/L YNB, 1.92 g/L yeast synthetic drop-out medium supplemented without uracil (Sigma-Aldrich, St. Louis, MO, USA), 80 mg/L uracil, and 20 g/L glucose], SCG medium (6.7 g/L YNB, 1.92 g/L yeast synthetic drop-out medium supplement without uracil, 80 mg/L uracil, and 20 g/L glycerol), YPD medium [10 g/L yeast extract, 20 g/L peptone, and 20 g/L glucose], or YPG medium (10 g/L yeast extract, 20 g/L peptone, and 20 g/L glycerol) at 30 °C and 200 rpm. These media were supplemented with appropriate antibiotics, including 300 mg/L hygromycin, 500 mg/L G418, and 100 mg/L Zeocin. For preparing plates, 20 g/L agar was added to the medium.

### Flask fermentation

Single colonies were inoculated into test tubes containing 5 mL of YPG or YPD medium supplemented with appropriate antibiotics at 200 rpm and 30 °C for preculture. After overnight cultivation, the yeast cells were washed with 500 μL distilled water and inoculated into a 100 mL Erlenmeyer flask containing 20 mL of YPG or YPD medium at an initial OD_600_ of 0.05 and grown for 96 h at 150 rpm and 30 °C. Culture samples were collected every 24 h to measure the cell growth (OD_600_) and concentrations of glycerol, glucose, *p*-coumarate, naringenin, and resveratrol in the culture medium.

### Jar fermentation

Yeast cells were precultured in a 100 mL Erlenmeyer flask containing 20 mL of SD, SG, SCD, or SCG medium supplemented with appropriate antibiotics at 150 rpm and 30 °C for 48 h. After preculture, the yeast cells were washed with 500 μL distilled water and inoculated into a 250 mL bioreactor (Bio Jr. 8; ABLE Biott, Tokyo, Japan) containing 100 mL of SD, SG, SCD, or SCG medium at an initial OD_600_ of 0.05. Fermentation was performed at 400 rpm and 30 °C, and the air flow rate was maintained at 100 mL/min. To control the pH at 6.0 during fermentation, 5 M ammonia solution was automatically added, and Antifoam SI (FUJIFILM Wako Pure Chemical, Osaka, Japan) was manually added when foaming occurred in the bioreactor. Culture samples were collected at appropriate times to measure the cell growth (OD_600_) and concentrations of glycerol, glucose, *p*-coumarate, naringenin, and resveratrol in the culture medium.

For fermentation with the addition of single amino acids, the precultured yeast cells were inoculated into a 250 mL bioreactor containing 100 mL of SG medium supplemented with 400 mg/L leucine, 80 mg/L isoleucine, 80 mg/L valine, 80 mg/L methionine, 80 mg/L asparagine, 80 mg/L aspartate, 80 mg/L arginine, 80 mg/L lysine, 80 mg/L glutamine, 80 mg/L glutamate, 80 mg/L histidine, 80 mg/L phenylalanine, 80 mg/L tyrosine, or 80 mg/L tryptophan. The amount of amino acid added was based on the concentrations of yeast synthetic dropout medium supplemented without uracil (Sigma-Aldrich). Other fermentation conditions were the same as those described above.

## Metabolites analysis

The concentrations of glucose and glycerol in the culture medium were determined using a high-performance liquid chromatography (HPLC) system (Shimadzu, Kyoto, Japan) equipped with an Aminex HPX-87H column (9 μm particle size, 7.8 mm × 300 mm; Bio-Rad, Hercules, CA, USA) using a RID-10A refractive index detector (Shimadzu). The temperature of the column oven was maintained at 65 °C and 5 mM H_2_SO_4_ was used as the mobile phase at a flow rate of 0.6 mL/min.

*p*-Coumarate, naringenin, and resveratrol were extracted and quantified as previously described (Kobayashi et al. 2021; Kumokita et al. 2022). For the extraction of *p*-coumarate, naringenin, and resveratrol, equal volumes of 99.5 % ethanol were mixed with the culture samples. After vortexing and centrifugation at 14,000 g and 4 °C for 1 min, the supernatants were analyzed using an HPLC system equipped with a Luna Omega PS C18 column (3 μm particle size, 4.6 mm × 150 mm; Phenomenex, Torrance, CA, USA) and an SPD-20A UV/VIS detector (Shimadzu).

Equal volumes of chloroform were added to the culture supernatant to extract the metabolites from the culture medium. After vortexing and centrifugation at 14,000 g and 4 °C for 5 min, the supernatants were purified using a 3KDa Amicon Ultra cut-off filter device (Amicon Ultra-3K, Merck, Darmstadt, Germany). After filtration, (+)-10-camphorsulfonic acid was added to the samples as an internal standard to a final concentration of 1 μM. Intracellular metabolites were extracted from yeast cells as described previously (Kato et al. 2012). Metabolites of the shikimate pathway and amino acids were analyzed using an LCMS-8060 quadrupole mass spectrometer (Shimadzu) equipped with a Discovery HS F5-3 column (3 μm particle size, 2.1 mm × 150 mm; Sigma-Aldrich). Glycolysis metabolites were analyzed on a 6460 Triple Quad LC/MS (Agilent Technologies, Palo Alto, CA) equipped with a Mastro C18 column (3 μm particle size, 2.1 mm × 150 mm; Shimadzu). Details of the operating conditions have been described previously (Hsu et al. 2017; Vavricka et al. 2019).

## Statistical analysis

Data are presented as mean ± standard deviation. Student’s *t*-test (unequal variance of two samples; two-tailed, *p < 0.05) was performed for statistical analysis using Microsoft Excel 2016 (Microsoft, Redmond, WA, USA).

## Results

### Comparison of aromatic secondary metabolites produced by *P. pastoris* using glucose or glycerol as a carbon source

**Fig. 1:**
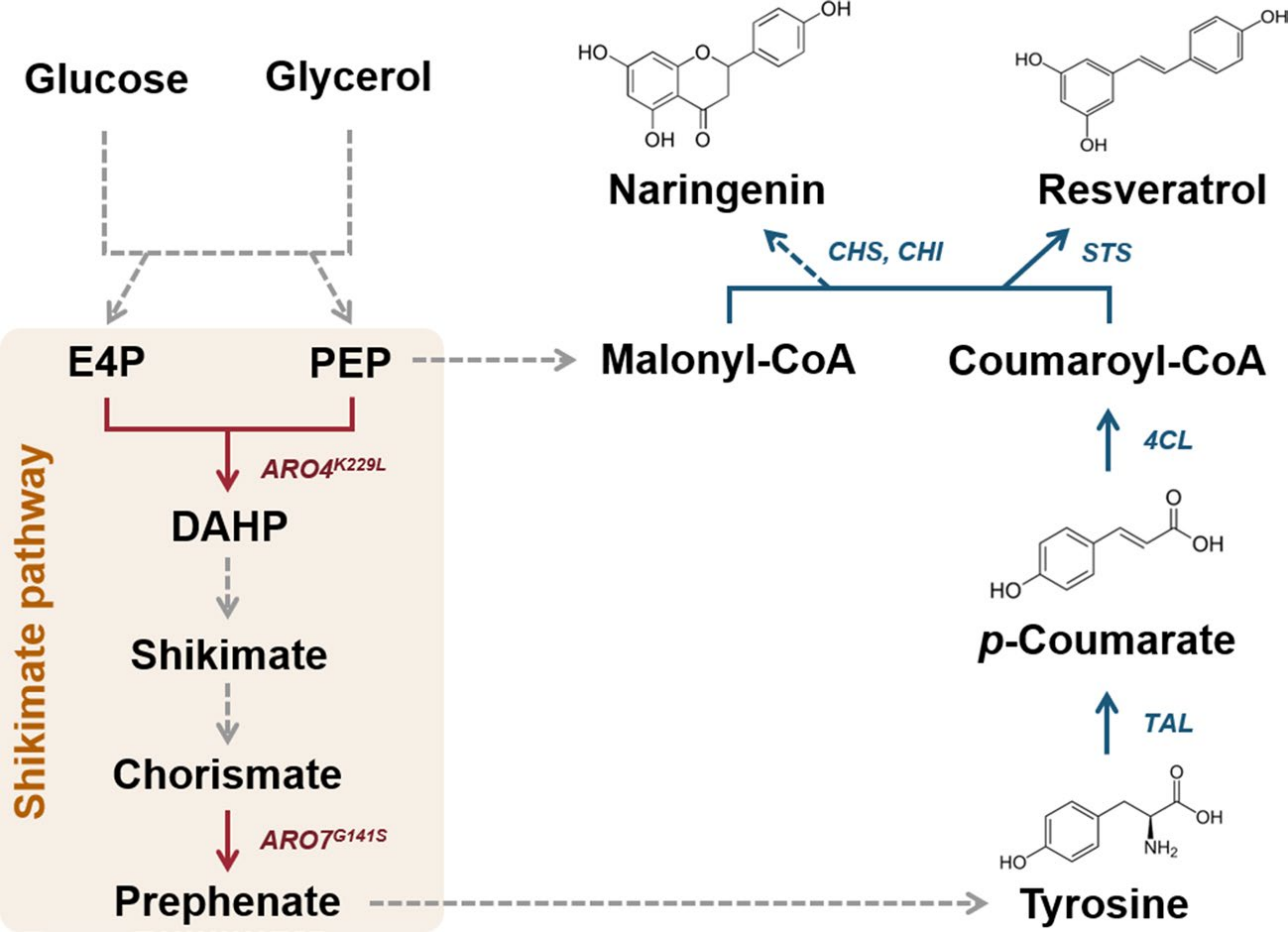
**Biosynthetic pathways of aromatic secondary metabolites in** ***Pichia pastoris***. Biosynthetic pathways of *p*-coumarate, naringenin, and resveratrol in *P. pastoris*. Gray and red arrows represent the native metabolic pathways in *P. pastoris*. The necessary heterologous pathways for producing *p*-coumarate, naringenin, and resveratrol are indicated by blue arrows. Multiple enzymatic steps are indicated by dashed arrows. PEP: phosphoenolpyruvate; E4P: erythrose-4-phosphate; DAHP: 3-deoxy-D-arabino-heptulosonate-7-phosphate; *ARO4*^*K229L*^: DAHP synthase (K229L); *ARO7*^*G141S*^: chorismate mutase (G141S); *TAL*: tyrosine ammonia-lyase; *4CL*: 4-coumarate CoA ligase; *STS*: stilbene synthase; *CHS*: chalcone synthase; *CHI*: chalcone isomerase

Aromatic secondary metabolites, including *p*-coumarate, naringenin, and resveratrol, are biosynthesized from the aromatic amino acid tyrosine (Fig. 1). In a previous study, we showed that the titers of aromatic secondary metabolites in *P. pastoris* significantly increased with the overexpression of genes encoding feedback inhibition resistant mutants of 3-deoxy-D-arabino-heptulosonate-7-phosphate synthase (*ARO4*^*K229L*^) and chorismate mutase (*ARO7*^*G141S*^) from *S. cerevisiae* to reduce feedback inhibition from the shikimate pathway (Kumokita et al. 2022). Therefore, the T-ARO47m, T4CC-ARO47m, and T4V-ARO47m strains overexpressing the biosynthetic pathways of *p*-coumarate, naringenin, and resveratrol, respectively, and further overexpressing *ARO4*^*K229L*^ and *ARO7*^*G141S*^ were used to compare the production of aromatic secondary metabolites by *P. pastoris* using glucose or glycerol as the carbon source.

**Fig. 2:**
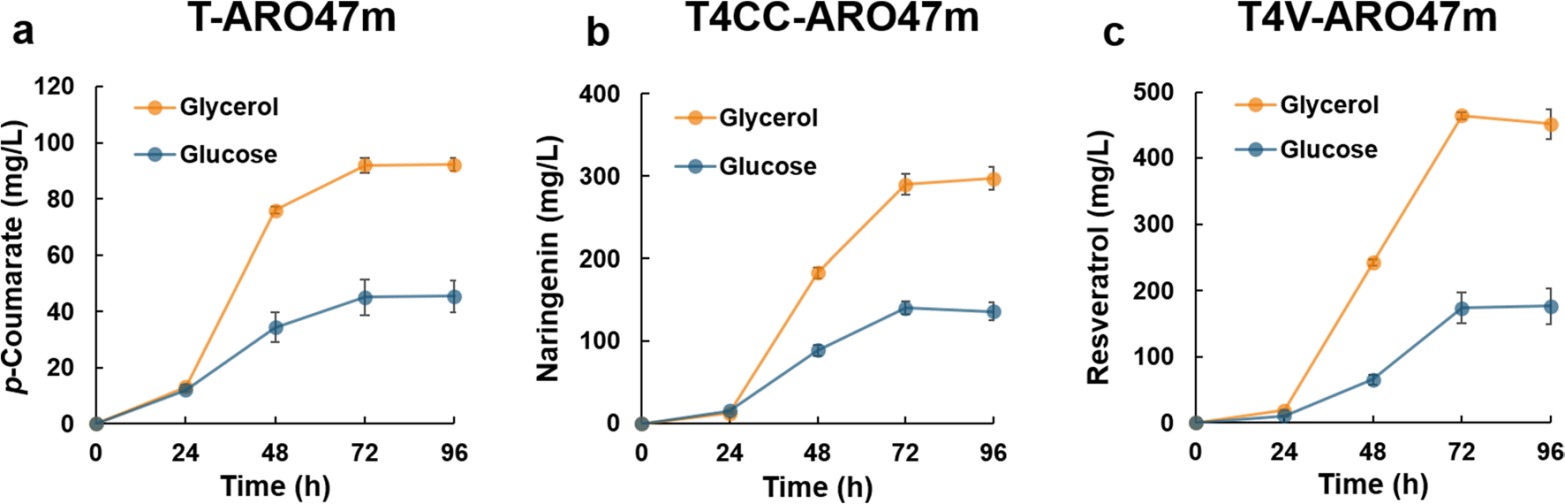
**Aromatic secondary metabolite production by** ***P. pastoris*** **using glycerol or glucose**. The *p*-coumarate-producing (T-ARO47m), naringenin-producing (T4CC-ARO47m), and resveratrol-producing (T4V-ARO47m) strains were cultivated in 100 mL Erlenmeyer flasks containing 20 mL of YPD (glucose) or YPG (glycerol) medium at 30 °C and 150 rpm. **(a–c)** Time course of *p*-coumarate, naringenin, and resveratrol production in T-ARO47m, T4CC-ARO47m, and T4V-ARO47m strains. Blue and orange lines represent the titers of aromatic secondary metabolites in YPD or YPG medium, respectively. Error bars represent as mean ± standard deviation of three independent biological samples

The *p*-coumarate-producing strain (T-ARO47m) produced 45 mg/L *p*-coumarate from 20 g/L glucose after 96 h of flask cultivation, but the titer increased by 103 % to 92 mg/L when cultivated with 20 g/L glycerol (Fig. 2a). The naringenin-producing strain (T4CC-ARO47m) produced 136 mg/L naringenin from 20 g/L glucose after 96 h of flask cultivation, but the titer increased by 118 % to 297 mg/L when cultivated with 20 g/L glycerol (Fig. 2b). The resveratrol-producing strain (T4V-ARO47m) produced 176 mg/L resveratrol from 20 g/L glucose after 96 h of flask cultivation, but the titer increased by 157 % to 452 mg/L when cultivated with 20 g/L glycerol (Fig. 2c). These results showed that the titers of aromatic secondary metabolites in *P. pastoris* varied greatly depending on the carbon source. Regarding the consumption of carbon source, each *P. pastoris* strain consumed 20 g/L glucose within 48 h and 20 g/L glycerol in over 96 h (Supplemental Fig. S1). During fermentation, cell growth (OD_600_) was not significantly different regardless of the carbon source (Supplemental Fig. S1).

### Aromatic secondary metabolite production using amino acids as carbon sources

Aromatic secondary metabolites production when amino acids were used as additional carbon sources was investigated. Aromatic secondary metabolites were produced in synthetic media with (SCD and SCG) or without (SD and SG) amino acids. The compositions of the individual media are described in the “Material and methods” section. These synthetic media contained ammonium sulfate as the nitrogen source; nitrification decreased the pH of the culture medium, which was detrimental to yeast cell growth (Libkind et al. 2004; Libkind and Van Broock 2006). Therefore, we used a bioreactor system in which the pH of the culture medium was maintained constant during fermentation.

**Fig. 3:**
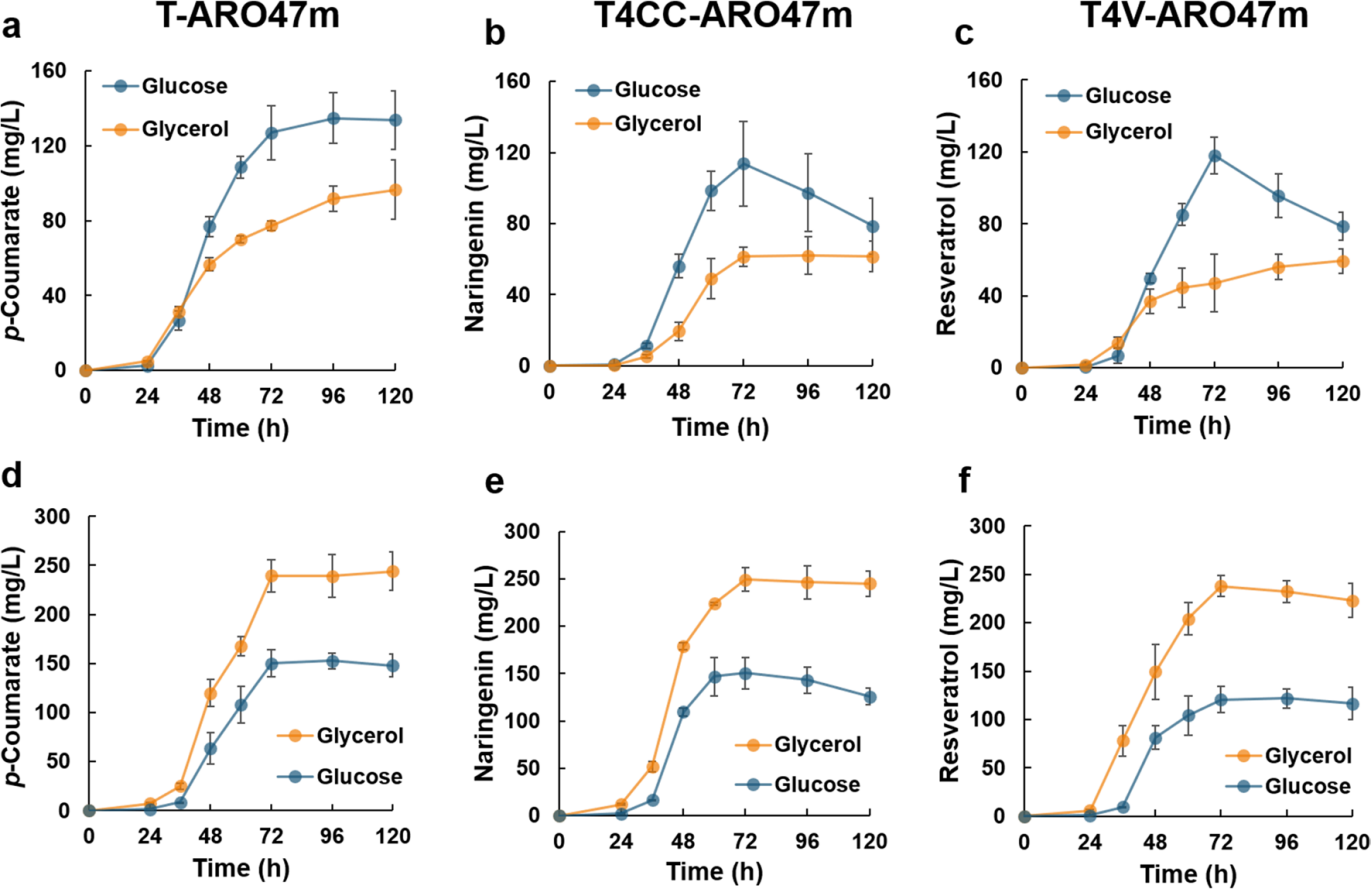
**Aromatic secondary metabolite production using amino acids as additional carbon sources**. The *p*-coumarate-producing (T-ARO47m), naringenin-producing (T4CC-ARO47m), and resveratrol-producing (T4V-ARO47m) strains were fermented in a 250 mL bioreactor (with a medium volume of 100 mL) at 30 °C, 400 rpm, and pH 6.0. **(a–c)** Time course of *p*-coumarate, naringenin, and resveratrol production in SD (glucose) or SG (glycerol) medium by T-ARO47m, T4CC-ARO47m, and T4V-ARO47m strains, respectively. Blue and orange lines represent the titers of aromatic secondary metabolites in SD or SG medium, respectively. **(d–f)** Time course of *p*-coumarate, naringenin, and resveratrol production in SCD (glucose) or SCG (glycerol) medium by T-ARO47m, T4CC-ARO47m, and T4V-ARO47m strains, respectively. Blue and orange lines represent the titers of aromatic secondary metabolites in SCD or SCG medium, respectively. Error bars represent as mean ± standard deviation of three independent biological samples

The aromatic secondary metabolites produced by the engineered *P. pastoris* strains (T-ARO47m, T4CC-ARO47m, and T4V-ARO47m) in SD, SG, SCD, and SCG media were measured. The titers were higher when glucose, instead of glycerol, was used as the carbon source during cultivation in the SD and SG media (Fig. 3a–c). By contrast, the titers were higher when glycerol, instead of glucose, was used as the carbon source during cultivation in the SCD and SCG media (Fig. 3d–f). Surprisingly, when cultured with glucose, the titers of *p*-coumarate, naringenin, and resveratrol were almost identical regardless of the presence or absence of amino acids in the culture medium (Fig. 3). By contrast, when cultured with glycerol, the titers of *p*-coumarate, naringenin, and resveratrol increased by 153 %, 298 %, and 275 %, respectively, after 120 h of fermentation in the SCG medium compared with the SG medium (Fig. 3). These results showed that *P. pastoris* could produce high levels of aromatic secondary metabolites from glycerol, but not from glucose, under amino acid-containing culture conditions. For carbon source consumption, the results were similar to those of flask fermentation, with 20 g/L glucose consumed by each engineered *P. pastoris* strain in 48 h, whereas 20 g/L glycerol was slowly consumed over 72–96 h (Supplemental Fig. S2). The cell growth (OD_600_) of each engineered *P. pastoris* strain did not differ between carbon sources, even when cultured in a bioreactor (Supplemental Fig. S3).

### Comprehensive time-course analysis of intracellular and extracellular metabolites

To elucidate the factors contributing to the differences in the titers of aromatic secondary metabolites during cultivation with glucose or glycerol, we performed a comprehensive time-course analysis of the intracellular and extracellular metabolites during fermentation on the SCD (glucose) or SCG (glycerol) medium for 30, 36, 42, 48, 60, and 72 h using a resveratrol-producing strain (T4V-ARO47m).

**Fig. 4:**
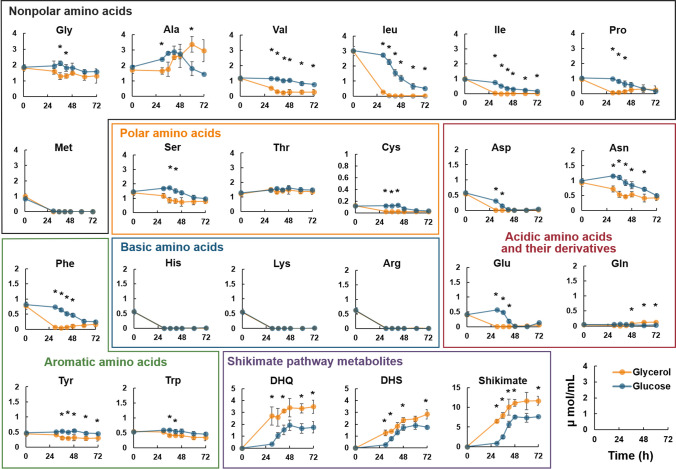
**Comparison of extracellular metabolites during fermentation of resveratrol producing strains grown in SCD or SCG medium**. The resveratrol-producing strain (T4V-ARO47m) was fermented in a 250 mL bioreactor containing 100 mL of the SCD (glucose) or SCG (glycerol) medium at 30 °C, 400 rpm, and pH 6.0. Extracellular metabolites produced during fermentation were determined through liquid chromatography-tandem mass spectrometry (LC-MS/MS). Blue and orange lines represent the concentrations of extracellular metabolites during fermentation in SCD or SCG medium, respectively. All units on the y-axis are μmol/mL. Error bars represent as mean ± standard deviation of three independent biological samples. Statistical analysis was performed using the Student’s *t*-test (unequal variance of two samples; two-tailed, *p < 0.05). Gly: glycine; Ala: alanine; Val: valine; Leu: leucine; Ile: isoleucine; Pro: proline; Met: methionine; Ser: serine; Thr: threonine; Cys: cysteine; His: histidine; Lys: lysine; Arg: arginine; Asp: aspartate; Asn: asparagine; Glu: glutamate; Gln: glutamine; Phe: phenylalanine; Tyr: tyrosine; Trp: tryptophan; DHQ: 3-dehydroquinate; DHS: 3-dehydroshikimate

The results of a comprehensive time-course analysis of intracellular and extracellular metabolites are shown in Supplemental Fig. S4 and Fig. 4, respectively. Several amino acids, including Met, His, Lys, and Arg, were depleted in the culture media after 30 h of fermentation in the SCD and SCG media (Fig. 4). The concentrations of many amino acids (Val, Leu, Ile, Asp, Asn, Glu, Phe, Tyr, and Trp) were lower in the SCG medium than in the SCD medium (Fig. 4), indicating that these amino acids were utilized by *P. pastoris* cells when grown with glycerol as a carbon source. By contrast, metabolites of the shikimate pathway, including 3-dehydroquinate (DHQ), 3-dehydroshikimate (DHS), and shikimate, were effluxed more when grown in the SCG medium than when grown in the SCD medium (Fig. 4). The intracellular concentrations of DHQ, DHS, and shikimate were also higher when cultured in the SCG medium than when cultured in the SCD medium (Supplemental Fig. S4).

### Fermentation with the addition of single amino acids

**Fig. 5:**
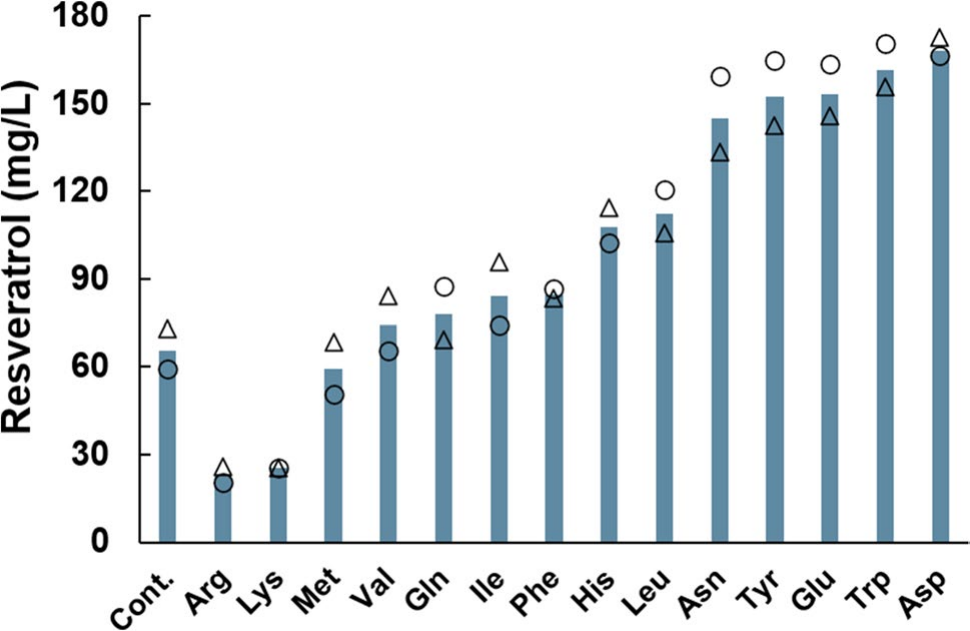
**Cultivation of resveratrol-producing strain in SG medium with the addition of single amino acids**. The resveratrol-producing strain (T4V-ARO47m) was fermented in a 250 mL bioreactor containing 100 mL of the SG medium with the addition of single amino acids at 30 °C, 400 rpm, and pH 6.0. Circles and triangles represent the resveratrol titers (*n* = 2) after 120 h of fermentation, and the values in the bar graph are the averages of the two samples. Cont. represents the results without the addition of amino acids (cultured in the SG medium). Arg: arginine; Lys: lysine; Met: methionine; Val: valine; Gln: glutamine; Ile: isoleucine; Phe: phenylalanine; His: histidine; Leu: leucine; Asn: asparagine; Tyr: tyrosine; Glu: glutamate; Trp: tryptophan; Asp: aspartate

We produced resveratrol using the T4V-ARO47m strain through fermentation with the addition of single amino acids. Based on the results of the comprehensive extracellular metabolite analysis shown in Fig. 4, we selected 14 amino acids for addition (Tyr, Phe, Trp, His, Met, Asn, Asp, Leu, Val, Ile, Gln, Glu, Arg, and Lys). These amino acids were those that were depleted or decreased in concentration when cultured in the SCG medium compared with the SCD medium. Interestingly, the titer of resveratrol greatly varied depending on the amino acid added (Figs. 5 and Supplemental Fig. S5a). For example, the resveratrol titers were reduced by more than half after 120 h of cultivation with Arg or Lys compared with those after the control fermentation (cultivation in SG medium) (Fig. 5). By contrast, the resveratrol titers increased by 121 %, 132 %, 134 %, 146 %, and 156 %, respectively, after cultivation with Asn, Tyr, Glu, Trp, and Asp compared with those after the control fermentation (Fig. 5). Consumption of the carbon source did not differ between the amino acid additions, and 20 g/L glycerol was consumed over 96 h under all conditions (Supplemental Fig. S5b).

**Fig. 6:**
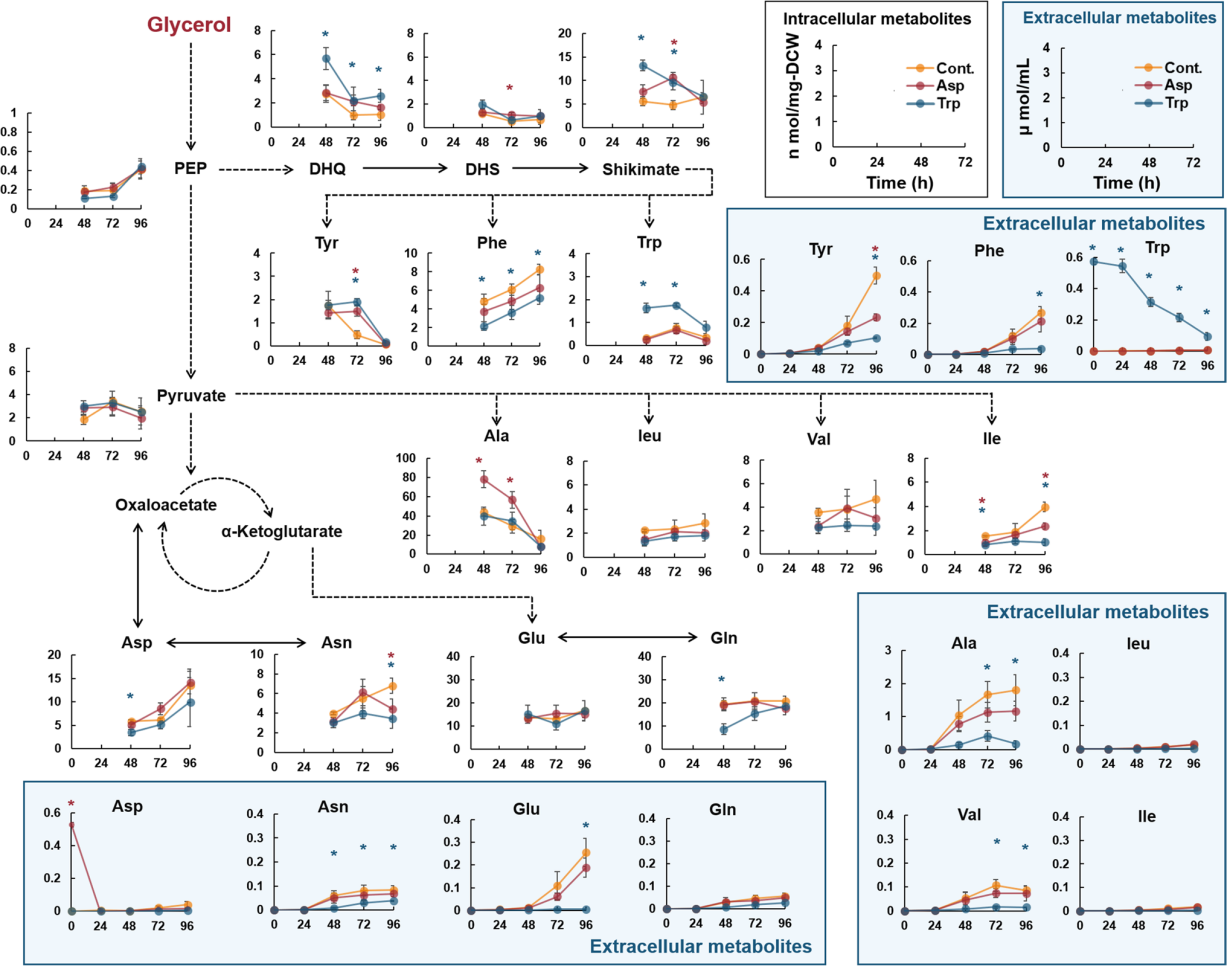
**Comparison of the concentration of intracellular and extracellular metabolites during fermentation of resveratrol-producing strain grown in SG medium supplemented with Asp or Trp**. The resveratrol-producing strain (T4V-ARO47m) was fermented in a 250 mL bioreactor containing 100 mL of SG medium supplemented with Asp or Trp at 30 °C, 400 rpm, and pH 6.0. Intracellular and extracellular metabolites produced during fermentation were determined using LC-MS/MS. Orange, red, and blue lines represent the concentrations of intracellular and extracellular metabolites during fermentation in SG (control), SG+Asp, and SG+Trp media, respectively. All units on the y-axis for figures showing intracellular metabolites are n-mol/mg dry cell weight (DCW). All units on the y-axis for figures showing extracellular metabolites are μmol/mL. Extracellular metabolites are represented by squares. Multiple enzymatic steps are indicated by dashed arrows. Error bars represent as mean ± standard deviation of three independent biological samples. Statistical analysis was performed using the Student’s *t*-test (unequal variance of two samples; two-tailed, *p < 0.05). Red and blue asterisks indicate statistically significant differences (*p < 0.05) between fermentation results under Asp or Trp conditions and in SG medium (control), respectively. PEP: phosphoenolpyruvate. DHQ: 3-dehydroquinate; DHS: 3-dehydroshikimate; Tyr: tyrosine; Phe: phenylalanine; Trp: tryptophan; Ala: alanine; Leu: leucine; Val: valine; Ile: isoleucine; Asp: aspartate; Asn; asparagine; Glu; glutamate; Gln; glutamine

Finally, a comprehensive time-course analysis of the intracellular and extracellular metabolites was performed during the cultivation in SG medium supplemented with Asp or Trp, the amino acids that increased resveratrol titers the most, as shown in Fig. 5. The concentrations of many amino acids (Asn, Glu, Ala, Val, Tyr, and Phe) in the culture medium were lower after cultivation with Asp or Trp than after the control fermentation (Fig. 6). By contrast, the intracellular concentrations of shikimate pathway metabolites, including DHQ, DHS, shikimate, and tyrosine, were higher after cultivation with Asp or Trp compared with those after the control fermentation (Fig. 6). The results of the comprehensive time-course analysis of the intracellular and extracellular metabolites are summarized in Supplemental Figs. S6 and S7, respectively. These results confirm that *P. pastoris* could increase the metabolic flux toward the production of aromatic secondary metabolites with enhanced amino acid utilization when Asp or Trp was supplemented in the medium. The results of this study provide valuable insights for improving aromatic secondary metabolite production by *P. pastoris*.

## Discussion

The yeast *P. pastoris* has been widely used for the industrial production of proteins for various applications (Barone et al. 2023). It has recently been investigated as a host for producing various chemicals and materials through metabolic engineering (Carneiro et al. 2022; Shrivastava et al. 2023). In previous studies, we showed that *P. pastoris* is an attractive host for producing aromatic secondary metabolites. In addition, the titer of resveratrol, an aromatic secondary metabolite, increased by 156 % when cultured with glycerol instead of glucose (Kumokita et al. 2022). In the present study, the titers of *p*-coumarate, naringenin, and resveratrol increased by 103 %, 118 %, and 157 %, respectively, when *P. pastoris* was cultivated with glycerol instead of glucose (Fig. 2a–c). Interesting results have been reported in other hosts for the production of aromatic secondary metabolites. For resveratrol production in *Scheffersomyces stipitis* in 200 mL flasks, 238 mg/L resveratrol was produced from 50 g/L glucose, whereas 669 mg/L resveratrol was produced from 50 g/L sucrose (Kobayashi et al. 2021). For resveratrol production in *Y. lipolytica* in 24 deep-well plates, 402 mg/L resveratrol was produced from 20 g/L glucose, whereas 145 mg/L resveratrol was produced from 20 g/L glycerol (Sáez-Sáez et al. 2020). The production of aromatic secondary metabolites varies widely among hosts and carbon sources, even when cultured in the same natural complex medium (containing 10 g/L yeast extract and 20 g/L peptone). Further studies, including metabolic flux analyses, are warranted to compare the metabolic mechanisms in different microbial hosts and the conditions suitable for aromatic secondary metabolite production.

Although *p*-coumarate is a precursor for the production of resveratrol and naringenin (Fig. 1), the titer of *p*-coumarate was lower than the titers of resveratrol and naringenin from both carbon sources (glucose and glycerol) during flask fermentation (Fig. 2). This may be due to a decrease in the pH of the culture medium as a result of *p*-coumarate production by *P. pastoris*, considering that *p*-coumarate is acidic. Indeed, no significant differences in the titers of *p*-coumarate, resveratrol, and naringenin were observed during pH-controlled jar fermentation (Fig. 3). Controlling the pH of the culture medium is essential for high production of acidic compounds. During cultivation on minimal synthetic medium with glucose but without amino acids (SD medium), the titers of naringenin and resveratrol decreased after 72 h of fermentation (Fig. 3b and 3c). Similar phenomena have been reported for resveratrol production from glucose in *C. glutamicum* and from molasses in *S. stipitis* (Braga et al. 2018; Kobayashi et al. 2022). Braga et al. suggested that the antioxidant resveratrol may be oxidized or oligomerized because of an increase in dissolved oxygen concentration following substrate depletion. In this study, similar phenomena may have occurred not only with resveratrol but also with another antioxidant, naringenin. However, cultivation with glycerol or glucose in an amino acid-containing medium did not decrease the resveratrol or naringenin titers (Figs. 2 and 3). The time required for glycerol depletion was longer than that required for glucose depletion (Supplemental Figs. S1 and S2), and under conditions with amino acids, amino acids were still present as an alternative carbon source even after glucose and glycerol were depleted. Therefore, the cells could utilize them as carbon sources, that is, they could consume oxygen through respiration, which might have prevented the oxidation or oligomerization of these aromatic secondary metabolites.

During cultivation in natural complex media, the titers of aromatic secondary metabolites were higher when cultured with glycerol than when cultured with glucose (Fig. 2). Similar results were obtained during cultivation on synthetic media supplemented with amino acids (SCD or SCG), suggesting the superiority of aromatic secondary metabolite production from glycerol by *P. pastoris* (Fig. 3d–f). By contrast, all titers decreased when *P. pastoris* was cultured with glycerol than when it was cultured with glucose in the minimal synthetic medium without amino acids (SD or SG) (Fig. 3a–c). Unlike glucose metabolism, glycerol metabolism does not provide sufficient reducing power or energy, which makes the biosynthesis of many intracellular metabolites, including amino acids, more difficult and consequently reduces their productivity. A comprehensive time-course analysis of the intracellular and extracellular metabolites during fermentation showed that the amino acids in the culture media were well-utilized by *P. pastoris* cells and that the metabolites of the shikimate pathway, including DHQ, DHS, and shikimate, accumulated in *P. pastoris* cells when cultured with glycerol (Figs. 4 and Supplemental Fig. S4). These results suggest that *P. pastoris* strains using glycerol as a carbon source could improve amino acid utilization and increase the metabolic flux toward the production of aromatic secondary metabolites. Furthermore, the titer of resveratrol produced from glycerol significantly increased when grown in SG medium supplemented with Asp or Trp (Fig. 5). In addition, a comprehensive time-course analysis of intracellular and extracellular metabolites during fermentation showed that the concentration of many amino acids was lower when cultivated in the medium with Asp or Trp than when cultivated in SG medium (Fig. 6). The intracellular concentrations of the shikimate pathway metabolites (including DHQ, DHS, shikimate) and Tyr were high with Asp or Trp supplementation (Fig. 6). These results confirm that *P. pastoris*, when grown with glycerol as a carbon source, can increase metabolic flux toward the production of aromatic secondary metabolites with enhanced specific amino acid utilization. On the contrary, resveratrol titers in the presence of Arg or Lys were significantly lower than those of the control (Fig. 5). Crépin et al. reported that the rate of amino acid consumption in *S. cerevisiae* varies for different amino acids and depends on the kinetic properties of each transporter, which are regulated by multiple transcription factors (Crépin et al. 2012). When Arg or Lys is added to the *P. pastoris* fermentation mixture, the transcription factors associated with the consumption of these amino acids might regulate the expression of several genes involved in the production of aromatic secondary metabolites. Further studies are required to test our hypothesis, and one approach could be the evaluation of the expression levels of genes involved in the production of aromatic secondary metabolites.

We used a resveratrol-producing strain (T4V-ARO47m) to perform a comprehensive time-course metabolite analysis and fermentation with the addition of single amino acids. Considering the similar production behaviors for *p*-coumarate, naringenin, and resveratrol, as shown in Figs. 2 and 3, similar results are expected for the production of other aromatic secondary metabolites by *P. pastoris*. In this study, we found that *P. pastoris* could produce high levels of aromatic secondary metabolites from glycerol with enhanced utilization of specific amino acids through a comprehensive time-course analysis of the intracellular and extracellular metabolites during fermentation. The titers of aromatic secondary metabolites produced from glycerol in *P. pastoris* could be further improved in the future by optimizing the combination and concentration of amino acids in the medium. This study should provide a basis for accelerating the production of aromatic secondary metabolites by *P. pastoris*.

### Supplementary information

ESM 1 (PDF 1485 kb)

## Data Availability

The data obtained and/or analyzed in this study are available from the corresponding author upon reasonable request.
