# Supplementary material for: Aromatic secondary metabolite production from glycerol was enhanced by amino acid addition in Pichia pastoris
Source: Appl Microbiol Biotechnol. 2023 Sep 27;107(24):7391–401. doi: 10.1007/s00253-023-12798-5 (PMC10656317; doi:10.1007/s00253-023-12798-5)
Supplement: Supplementary file 1 — (PDF 1485 kb) [file 253_2023_12798_MOESM1_ESM.pdf]

# Supplementary Information

**Aromatic secondary metabolite production from glycerol was enhanced by amino acid addition in *Pichia pastoris***


Ryota Kumokita[1], Takanobu Yoshida[1], Tomokazu Shirai[1,2], Akihiko Kondo[1,2,3], Tomohisa Hasunuma[1,2,3*]

[1]Graduate School of Science, Technology and Innovation, Kobe University, 1-1 Rokkodai, Nada, Kobe 657-8501, Japan

[2]RIKEN Center for Sustainable Resource Science, 1-7-22 Suehiro, Tsurumi, Yokohama 230-0045, Japan

[3]Engineering Biology Research Center, Kobe University, 1-1 Rokkodai, Nada, Kobe, 657-8501, Japan

**Corresponding author:**

Tomohisa Hasunuma

Engineering Biology Research Center, Kobe University, 1-1 Rokkodai, Nada, Kobe, 657-8501, Japan; orcid.org/0000-0002-8382-2362

Telephone: +81-78-803-6461; Fax: +81-78-803-6461

E-mail: hasunuma@port.kobe-u.ac.jp

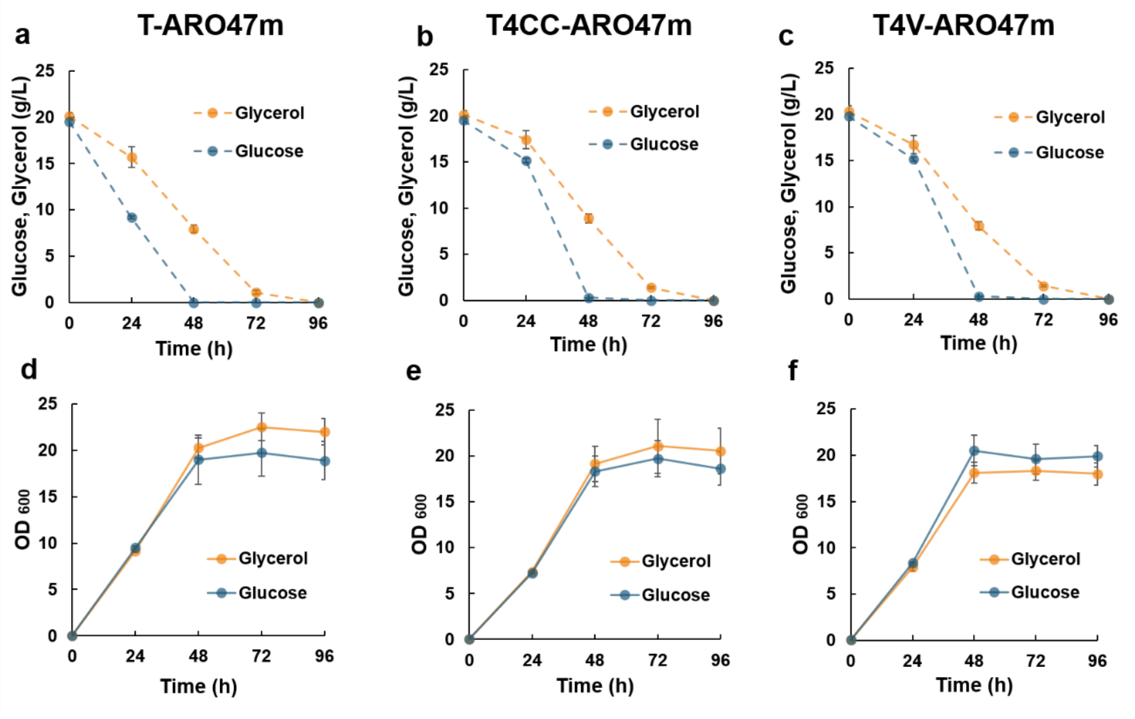


**Supplementary Figure S1**

**Glucose and glycerol consumption profiles and growth curve during cultivation in YPD or YPG medium**

The *p*-coumarate-producing (T-ARO47m), naringenin-producing (T4CC-ARO47m), and resveratrol-producing (T4V-ARO47m) strains were cultivated in 100 mL Erlenmeyer flasks containing 20 mL of YPD (glucose) or YPG (glycerol) medium at 30 °C and 150 rpm. **(a–c)** Glucose and glycerol consumption profiles of the T-ARO47m, T4CC-ARO47m, and T4V-ARO47m strains, respectively. Blue and orange dashed lines represent the concentrations of glucose and glycerol in YPD or YPG medium, respectively. **(d–f)** Growth curve of the T-ARO47m, T4CC-ARO47m, and T4V-ARO47m strains, respectively. Blue and orange lines represent the cell growth in YPD or YPG medium, respectively. Error bars represent as mean ± standard deviation of three independent biological samples

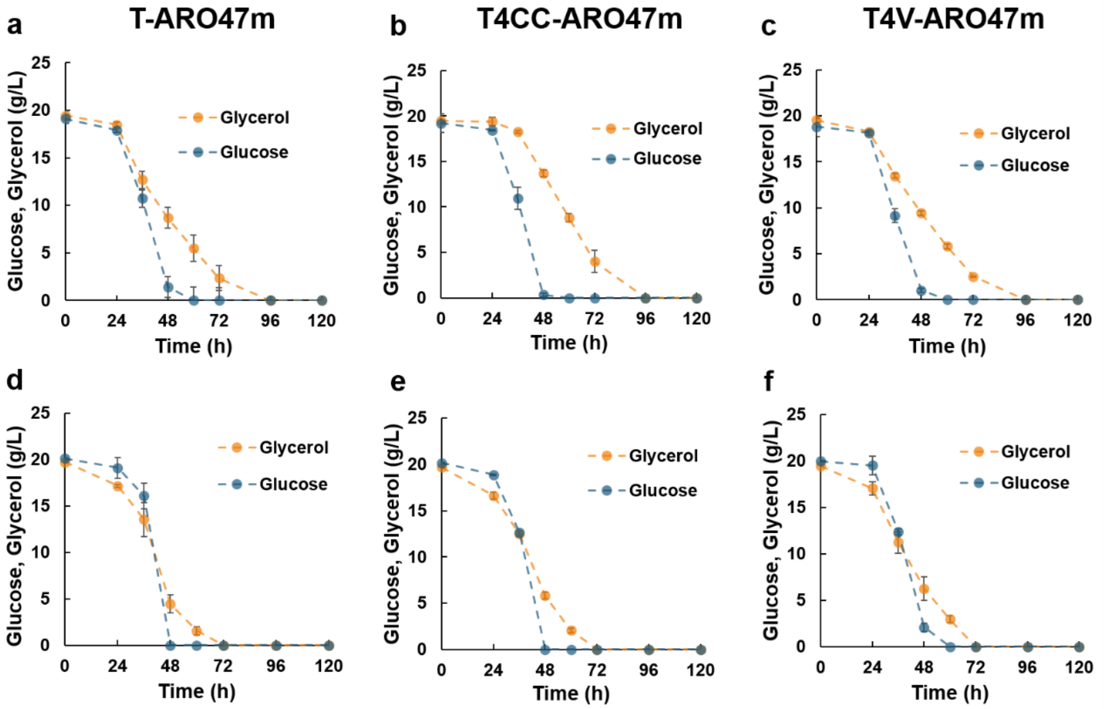


**Supplementary Figure S2**

**Glucose and glycerol consumption profiles during cultivation in SD, SG, SCD, or SCG medium**

The *p*-coumarate-producing (T-ARO47m), naringenin-producing (T4CC-ARO47m), and resveratrol-producing (T4V-ARO47m) strains were fermented in a 250 mL bioreactor (with a medium volume of 100 mL) at 30 °C, 400 rpm, and pH 6.0. **(a–c)** Glucose and glycerol consumption profiles of the T-ARO47m, T4CC-ARO47m, and T4V-ARO47m strains in SD (glucose) or SG (glycerol) medium, respectively. Blue and orange dashed lines represent the concentrations of glucose and glycerol in SD or SG medium, respectively. **(d–f)** Glucose and glycerol consumption profiles of the T-ARO47m, T4CC-ARO47m, and T4V-ARO47m strains in SCD (glucose) or SCG (glycerol) medium, respectively. Blue and orange dashed lines represent the concentrations of glucose and glycerol in SCD (glucose) or SCG (glycerol) medium, respectively. Error bars represent as mean ± standard deviation of three independent biological samples

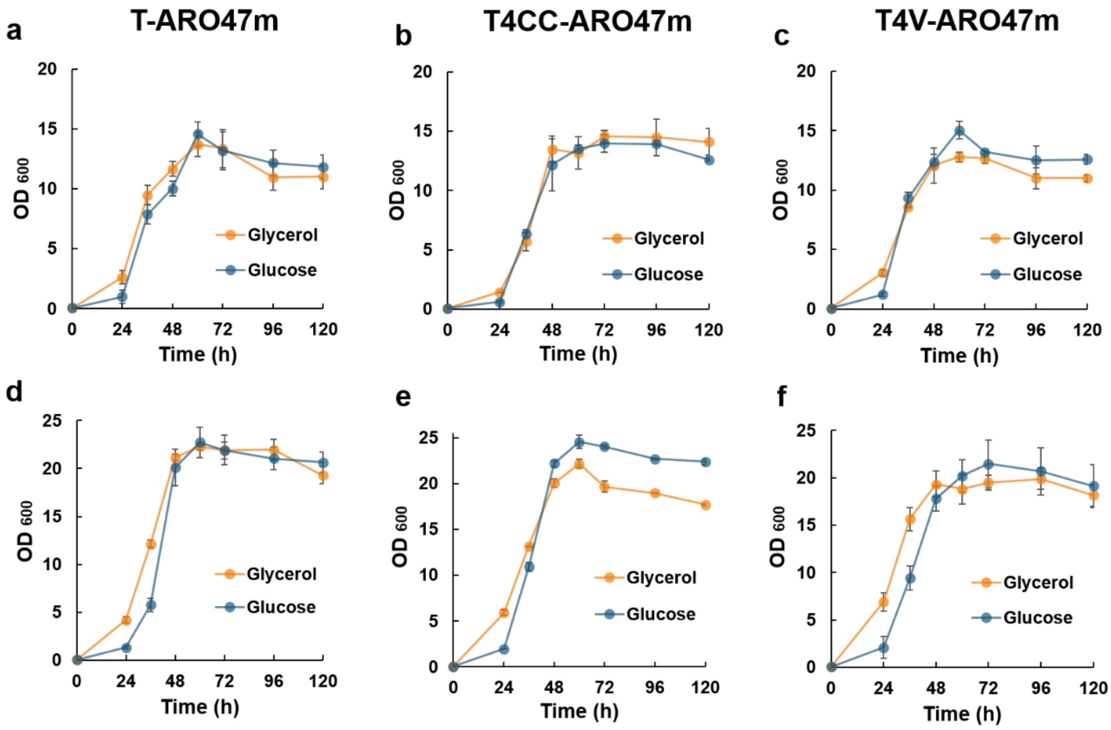


**Supplementary Figure S3**

**Growth curve during cultivation in SD, SG, SCD, or SCG medium**

The *p*-coumarate-producing (T-ARO47m), naringenin-producing (T4CC-ARO47m), and resveratrol-producing (T4V-ARO47m) strains were fermented in a 250 mL bioreactor (with a medium volume of 100 mL) at 30 °C, 400 rpm, and pH 6.0. **(a–c)** Growth curve of the T-ARO47m, T4CC-ARO47m, and T4V-ARO47m strains in SD (glucose) or SG (glycerol) medium, respectively. Blue and orange lines represent the cell growth in SD or SG medium, respectively. **(a–c)** Growth curve of the T-ARO47m, T4CC-ARO47m, and T4V-ARO47m strains in SCD (glucose) or SCG (glycerol) medium, respectively. Blue and orange lines represent the cell growth in SCD or SCG medium, respectively. Error bars represent as mean ± standard deviation of three independent biological samples

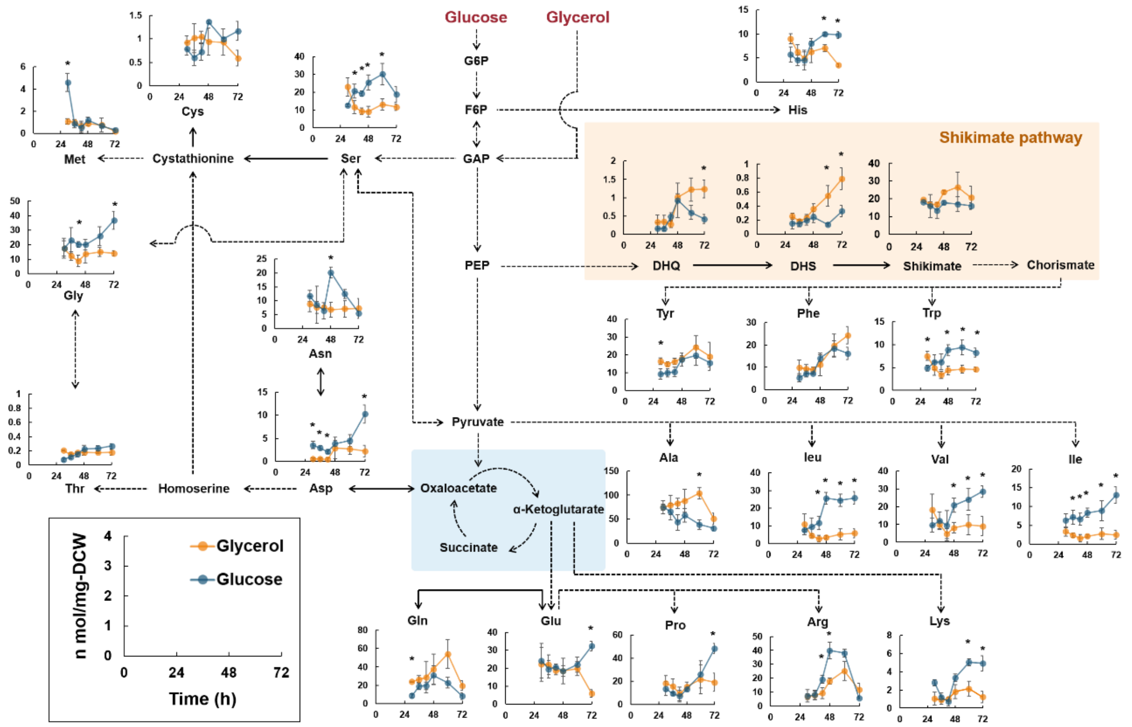


## Supplementary Figure S4

**Comparison of intracellular metabolites during fermentation of resveratrol producing strains grown in SCD or SCG medium**

The resveratrol-producing strain (T4V-ARO47m) was fermented in a 250 mL bioreactor containing 100 mL of the SCD (glucose) or SCG (glycerol) medium at 30 °C, 400 rpm, and pH 6.0. Intracellular metabolites produced during fermentation were determined through liquid chromatography-tandem mass spectrometry (LC-MS/MS). Blue and orange lines represent the concentrations of intracellular metabolites during fermentation in SCD or SCG medium, respectively. All units on the y-axis are n-mol/mg-dry cell weight (DCW). Multiple enzymatic steps are indicated by dashed arrows. Error bars represent as mean ± standard deviation of three independent biological samples. Statistical analysis was performed using the Student's *t*-test (unequal variance of two samples; two-tailed, $*p < 0.05$).

G6P: glucose-6-phosphate; F6P: fructose-6-phosphate; GAP: glyceraldehyde 3-phosphate; PEP: phosphoenolpyruvate; DHQ: 3-dehydroquinate; DHS: 3-dehydroshikimate; Tyr: tyrosine; Phe: phenylalanine; Trp: tryptophan; His: histidine; Ser: serine; Cys: cysteine; Met: methionine; Gly: glycine; Thr: threonine; Asn: asparagine; Asp: aspartate; Ala: alanine; Leu: leucine; Val: valine; Ile: isoleucine; Gln: glutamine; Glu: glutamate; Pro: proline; Arg: arginine; Lys: lysine

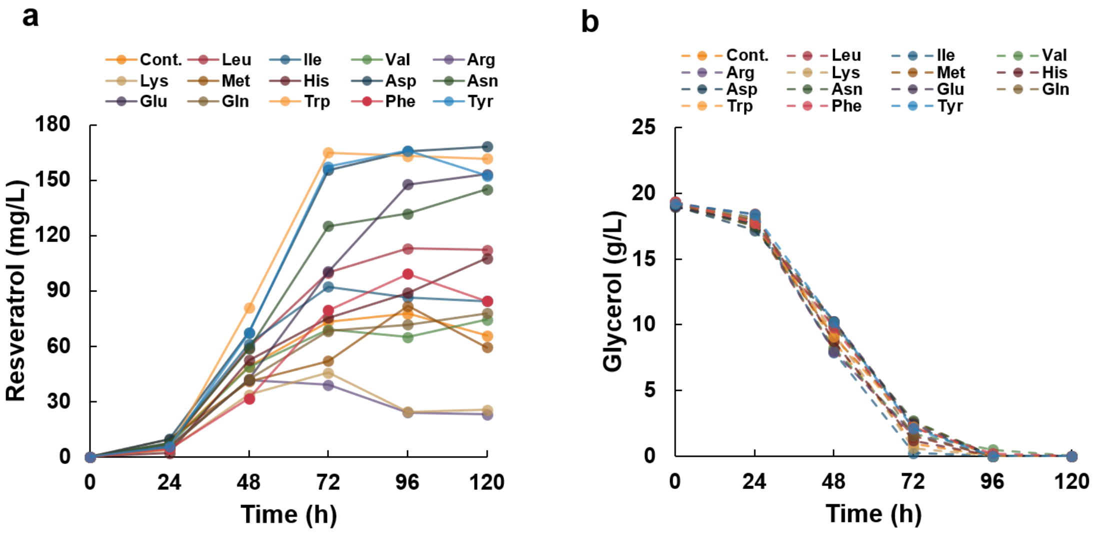


**Supplementary Figure S5**

**Time course of resveratrol production and glycerol consumption profiles in SG medium with the addition of single amino acids**

The resveratrol-producing strain (T4V-ARO47m) was fermented in a 250 mL bioreactor containing 100 mL of the SG medium with the addition of single amino acids at 30 °C, 400 rpm, and pH 6.0. (**a)** Time course of resveratrol production in SG medium with the addition of single amino acids by T4V-ARO47m strain. **b)** Glycerol consumption profiles of T4V-ARO47m strain in SG medium with the addition of single amino acids. The fermentation was performed twice and the values in these figures are the averages of the two samples. Cont. represents the results without the addition of amino acids (cultured in the SG medium). Arg: arginine; Lys: lysine; Met: methionine; Val: valine; Gln: glutamine; Ile: isoleucine; Phe: phenylalanine; His: histidine; Leu: leucine; Asn: asparagine; Tyr: tyrosine; Glu: glutamate; Trp: tryptophan; Asp: aspartate

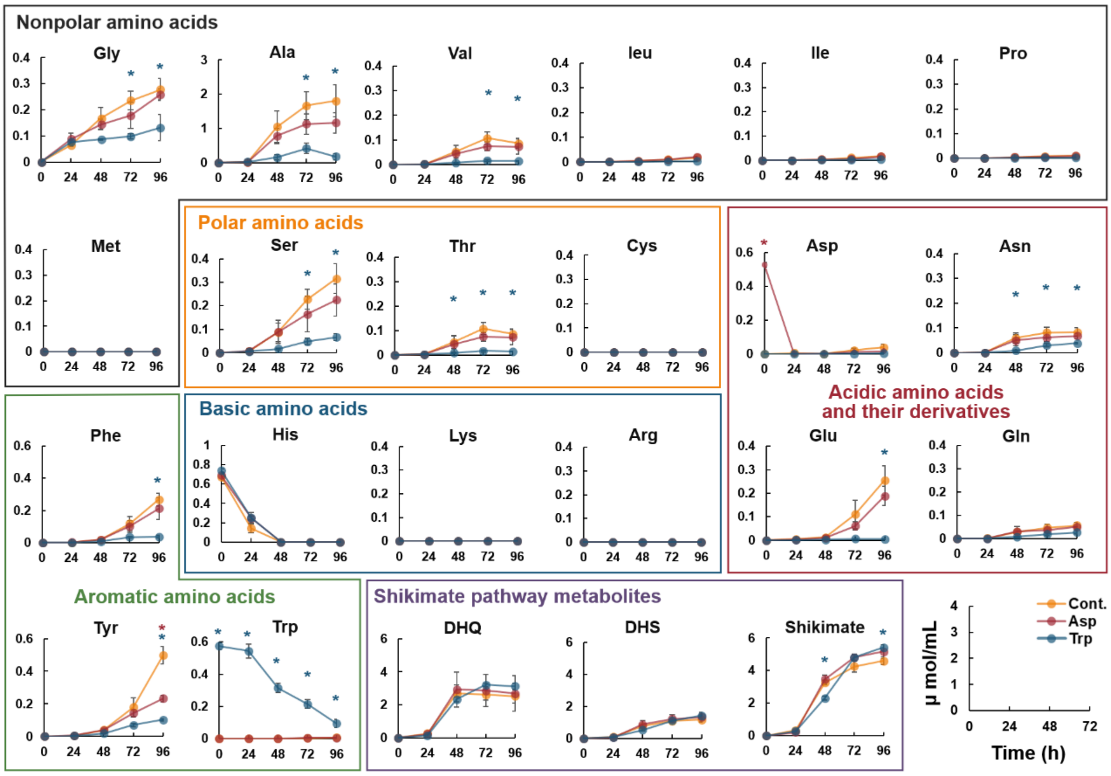


**Supplementary Figure S6**

**Comparison of intracellular metabolites during fermentation of resveratrol-producing strain grown in SG medium supplemented with Asp or Trp**

The resveratrol-producing strain (T4V-ARO47m) was fermented in a 250 mL bioreactor containing 100 mL of SG medium supplemented with Asp or Trp at 30 °C, 400 rpm, and pH 6.0. Intracellular metabolites produced during fermentation were determined using LC-MS/MS. Orange, red, and blue lines represent the concentrations of intracellular metabolites during fermentation in SG (control), SG+Asp, and SG+Trp media, respectively. All units on the y-axis are n-mol/mg-DCW. Multiple enzymatic steps are indicated by dashed arrows. Error bars represent as mean ± standard deviation of three independent biological samples. Statistical analysis was performed using the Student's *t*-test (unequal variance of two samples; two-tailed, *$p < 0.05$). Red and blue asterisks

indicate statistically significant differences (*$p < 0.05$) between fermentation results under Asp or Trp conditions and in SG medium (control), respectively.

F6P: fructose-6-phosphate; GAP: glyceraldehyde 3-phosphate; PEP: phosphoenolpyruvate; DHQ: 3-dehydroquinate; DHS: 3-dehydroshikimate; Tyr: tyrosine; Phe: phenylalanine; Trp: tryptophan; His: histidine; Ser: serine; Cys: cysteine; Met: methionine; Gly: glycine; Thr: threonine; Asn: asparagine; Asp: aspartate; Ala: alanine; Leu: leucine; Val: valine; Ile: isoleucine; Gln: glutamine; Glu: glutamate; Pro: proline; Arg: arginine; Lys: lysine

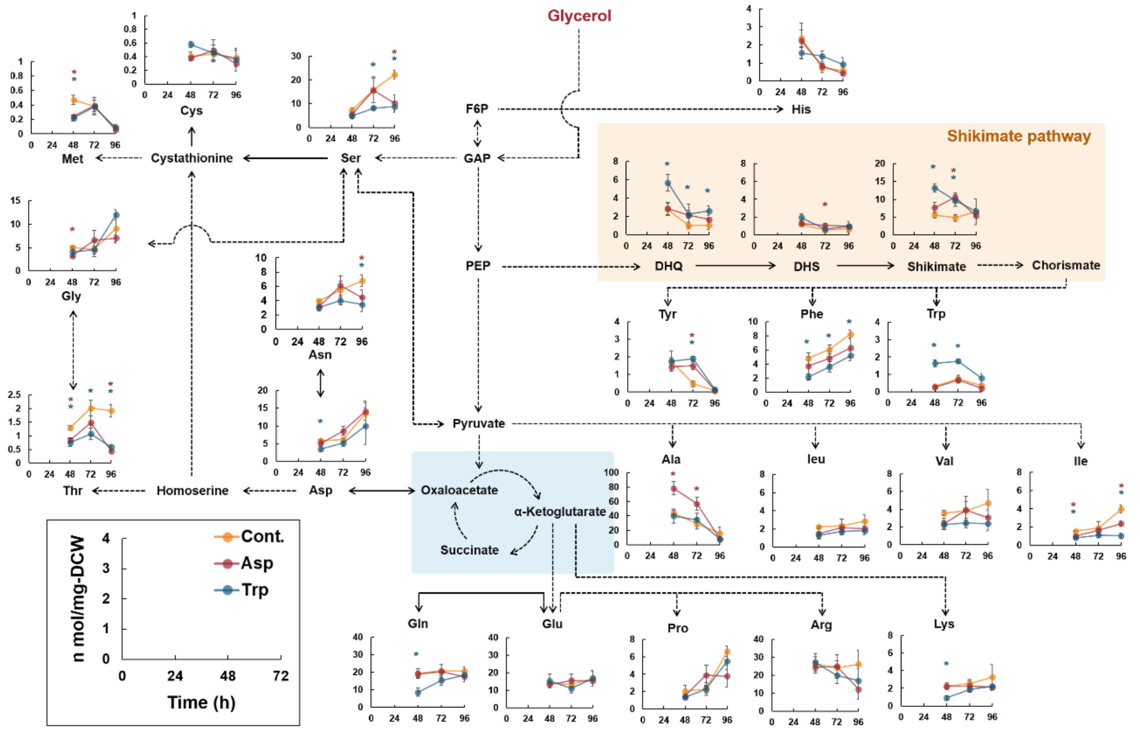


**Fig. S7 Comparison of extracellular metabolites during fermentation of resveratrol-producing strain grown in SG medium supplemented with Asp or Trp**

The resveratrol-producing strain (T4V-ARO47m) was fermented in a 250 mL bioreactor containing 100 mL of SG medium supplemented with Asp or Trp at 30 °C, 400 rpm, and pH 6.0. Extracellular metabolites produced during fermentation were determined using LC-MS/MS. Orange, red, and blue lines represent the concentrations of extracellular metabolites during fermentation in SG (control), SG+Asp, and SG+Trp media, respectively. All units on the y-axis are µmol/mL. Error bars represent as mean ± standard deviation of three independent biological samples. Statistical analysis was performed using the Student's *t*-test (unequal variance of two samples; two-tailed, $*p < 0.05$). Red and blue asterisks indicate statistically significant differences ($*p < 0.05$) between fermentation results under Asp or Trp conditions and in SG medium (control), respectively.

Gly: glycine; Ala: alanine; Val: valine; Leu: leucine; Ile: isoleucine; Pro: proline; Met: methionine; Ser: serine; Thr: threonine; Cys: cysteine; His: histidine; Lys: lysine; Arg: arginine; Asp: aspartate; Asn: asparagine; Glu: glutamate; Gln: glutamine; Phe: phenylalanine; Tyr: tyrosine; Trp: tryptophan; DHQ: 3-dehydroquinate; DHS: 3-dehydroshikimate
